# Basic symptoms in the community: prevalence, clinical significance and course

**DOI:** 10.1192/j.eurpsy.2025.178

**Published:** 2025-08-26

**Authors:** C. Michel, F. Schultze-Lutter

**Affiliations:** 1 University Hospital of Child and Adolescent Psychiatry and Psychotherapy, University of Bern, Bern, Switzerland; 2 Department of Psychiatry and Psychotherapy, Medical Faculty, Heinrich-Heine-University Düsseldorf, Düsseldorf, Germany

## Abstract

**Abstract: Objectives:**

The subtle, subjective nature of basic symptoms (BS) has often led to doubt regarding their clinical significance.

**Methods:**

We therefore examined the prevalence, clinical significance and course of BS over three years in a large sample (N = 2684) of 16- to 40-year-old residents of the Swiss canton of Bern (response rate: 64%). At follow-up, persons with a lifetime risk symptom at baseline were compared with control subjects (N = 834; response rate 66%). Fourteen criteria-relevant BSs were assessed by trained clinical psychologists over the telephone, along with information on symptomatic ultra-high-risk symptoms, mental disorders and functioning.

**Results:**

At baseline, 18% of the participants reported any lifetime BS, 10% had still experienced one in the three months prior to the interview. In general, BS were rare, and only 2% of the participants met any BS criteria, which was significantly associated with non-psychotic mental disorders (OR = 5) and especially with functional deficits (OR = 16). At follow-up, five individuals had developed psychosis, one with BS criteria and three more with BS at baseline. In addition, 95% of the participants no longer met the BS criteria, while 3% reported new BS criteria.

**Conclusions:**

Although BS are not rare phenomena in the community, they rarely persist and rarely occur frequently enough to meet the requirements of the risk criteria. Furthermore, they do not appear to occur randomly, but are restricted to a subpopulation of vulnerable individuals – possibly occurring in times of stress and/or low mood and functional difficulties in these individuals.

**Disclosure of Interest:**

None Declared

